# Heat stress-responsive transcriptome analysis in heat susceptible and tolerant wheat (*Triticum aestivum *L.) by using Wheat Genome Array

**DOI:** 10.1186/1471-2164-9-432

**Published:** 2008-09-22

**Authors:** Dandan Qin, Haiyan Wu, Huiru Peng, Yingyin Yao, Zhongfu Ni, Zhenxing Li, Chunlei Zhou, Qixin Sun

**Affiliations:** 1 Department of Plant Genetics & Breeding and State Key Laboratory for Agrobiotechnology, China Agricultural University, Beijing100193, PR China; 2 Key Laboratory of Crop Heterosis and Utilization (MOE), Key Laboratory of Crop Genomics and Genetic Improvement (MOA) and Beijing Key Laboratory of Crop Genetic Improvement, China Agricultural University, Beijing100193, PR China

## Abstract

**Background:**

Wheat is a major crop in the world, and the high temperature stress can reduce the yield of wheat by as much as 15%. The molecular changes in response to heat stress are poorly understood. Using GeneChip^® ^Wheat Genome Array, we analyzed genome-wide gene expression profiles in the leaves of two wheat genotypes, namely, heat susceptible 'Chinese Spring' (CS) and heat tolerant 'TAM107' (TAM).

**Results:**

A total of 6560 (~10.7%) probe sets displayed 2-fold or more changes in expression in at least one heat treatment (false discovery rate, FDR, α = 0.001). Except for heat shock protein (HSP) and heat shock factor (HSF) genes, these putative heat responsive genes encode transcription factors and proteins involved in phytohormone biosynthesis/signaling, calcium and sugar signal pathways, RNA metabolism, ribosomal proteins, primary and secondary metabolisms, as well as proteins related to other stresses. A total of 313 probe sets were differentially expressed between the two genotypes, which could be responsible for the difference in heat tolerance of the two genotypes. Moreover, 1314 were differentially expressed between the heat treatments with and without pre-acclimation, and 4533 were differentially expressed between short and prolonged heat treatments.

**Conclusion:**

The differences in heat tolerance in different wheat genotypes may be associated with multiple processes and mechanisms involving HSPs, transcription factors, and other stress related genes. Heat acclimation has little effects on gene expression under prolonged treatments but affects gene expression in wheat under short-term heat stress. The heat stress responsive genes identified in this study will facilitate our understanding of molecular basis for heat tolerance in different wheat genotypes and future improvement of heat tolerance in wheat and other cereals.

## Background

High temperature is one of the limiting factors affecting crop production. Combining with drought stress, the elevated temperature often causes yield loss and reduces the quality of crops [1-3]. Indeed, the high temperatures during the post-heading stages affect yield [4,5] and grain quality [6] of wheat, a major crop cultivated worldwide.

Plants can grow in the temperature above the optimal level that is known as basal thermotolerance. If plants are pretreated with a mild non-lethal temperature (heat acclimation) or if temperature increases gradually to a lethal level, they can survive under the lethal high temperature stress, which is known as acquired thermotolerance [7,8]. However, little is known about the molecular changes affecting regulatory and biochemical pathways of heat stress responses in crops [9]. Thus, identifying novel genes and studying their expression patterns in response to heat stress will provide a molecular basis for improving heat tolerance in crops.

Microarray analysis of gene expression has been used to investigate transcriptome changes in response to heat stress as well as combined stresses in several plant species, such as *Arabidopsis thaliana *[10], Chinese cabbage [9], *Festuca *[11] and barley [12]. Gene expression changes in wheat seedlings exposed to heat stress were analyzed using Affymetrix Barley1 Genechip [13], and analysis of ESTs (expressed sequence tags) was used to screen for the heat stress responsive genes in wheat [14]. To study gene expression changes in wheat related to basal and acquired thermotolerance, we used two wheat genotypes with different tolerance to heat stress for gene expression studies. 'Chinese Spring' (CS) is susceptible to heat stress, whereas 'TAM107' (TAM) is tolerant to heat stress. GeneChip^® ^Wheat Genome Array was applied to determine transcriptome changes in response to heat stress in these two genotypes. We identified a total of 6560 probe sets that were responsive to at least one heat treatment.

## Results and discussion

### Expressed probe sets in wheat leaves

**Figure 1:**
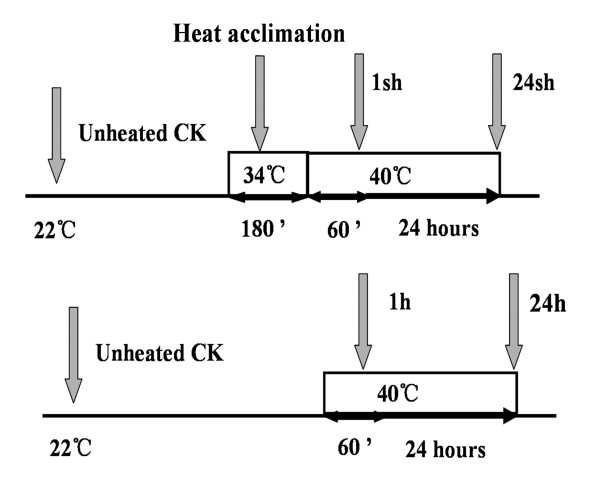
Heat treatments for comparison of acclimated and non-acclimated plants of the two genotypes.

The Affymetrix GeneChip^® ^Wheat Genome Array contains 61,127 probe sets, representing 55,052 transcripts (Affymetrix, USA). In this study, two wheat genotypes were used for expression analysis under heat treatments. Based on the cell membrane stability test, genotypes with higher relative injury (RI) value are heat susceptible, whereas genotypes with low RI value are heat tolerant [15]. 'Chinese Spring' (CS) is heat susceptible with a RI of 80%, and TAM107 (TAM) is heat tolerant with a RI of 35%. Ten-day-old seedlings were used for different heat treatments (Figure 1), resulting in four heat treatments and one control for each genotype (for detail, see Methods). Seedling leaves were harvested and used for microarray analysis using three biological replications, and a total of 30 arrays were hybridized. The normalized signal intensity of each sample was used to evaluate the reproducibility between three replications, and the correlation coefficients between any two replications ranged from 0.956 to 0.994, indicating that microarray analysis was highly reproducible in this study (See Additional file 1).

'Present' probe sets were detected by filtering with a fraction call 100% (for detail, see Methods). Those probe sets that were detected as 'Present' in all the three biological replications were defined as 'expressed' probe sets in this study. Based on these criteria, 32% and 30% of the 61,127 probe sets on the array were expressed in CS and TAM, respectively. The percentages of expressed probe sets were 26% for CS1h and 25% for TAM1h after short heat treatments, whereas the percentages increased in the long heat treatments (35% for CS24sh, 36% for CS24h, 34% for TAM24sh and 34% for TAM24h) (Table 1). The percentages in the short heat treatments with pre-heat acclimation were similar to those of the controls (Table 1). The data suggest that more genes were expressed when plants were treated under long heat stress (24 hours).

**Table 1:** Percentage of expressed probe sets and number of heat responsive probe sets in two wheat genotypes 'CS' and 'TAM107'.

	Chinese Spring				TAM107			
		
Sample	Expressed (%)	up	down	Total	Expressed (%)	up	down	Total
ck	31.78				29.67			
1 sh	30.30	1257	942	2199	30.35	1226	858	2084
1 h	26.01	1347	1271	2618	25.23	1509	1471	2980
24 sh	35.02	2361	1037	3398	34.21	1996	885	2881
24 h	36.27	2533	1097	3630	34.31	2068	862	2930

### Analysis of heat – responsive (HR) probe sets

#### Identification and validation of HR probe sets

The probe sets that were up- or down-regulated under heat stress in each treatment compared to the controls were referred to heat responsive (HR) probe sets. The fold changes should be equal or greater than 2 (false discovery rate, α = 0.001). A total of 6560 probe sets were identified to be heat responsive in at least one stress treatment (See Additional file 2), which represented 10.7% of the total probe sets on the array. In *Arabidopsis*, 11% of the genes showed significant expression changes after a 1-hour heat treatment [16], whereas 2.6% were responsive to a 6-hour heat treatment [10]. The discrepancy of the number of HR genes in these studies might result from the different species studied, the developmental stages when heat stress was applied, temperature and duration of heat treatments, and/or criteria of selecting candidate HR genes.

In this experiment, 1 h had more candidate HR probe sets than 1 sh treatments, and the latter resulted in the least number of HR probe sets (2199 for CS and 2084 for TAM) (Table 1). Pre-heat acclimation prior to the one-hour heat treatment reduced the number of HR probe sets, which could be important to protect wheat seedlings against otherwise lethal exposure to high temperature. It may also suggest that many early heat responsive genes, such as those encoding heat shock proteins (HSPs), heat shock factors (HSFs), and other transcription factors, are turned on during heat acclimation (34°C for three hours). Notably, approximately 70% of HR probe sets in 24 sh and 24 h treatments were up-regulated in both genotypes, whereas only 58% and 50% in 1 sh and 1 h treatments were up-regulated in CS and TAM, respectively (Table 1). More HR probe sets were detected in the heat susceptible 'CS' than in the heat tolerant wheat 'TAM107' (Table 1), indicating that the response of heat susceptible wheat genotype to long heat stress is reflected in gene expression changes.

To validate the expression levels of candidate HR genes detected by microarray analysis, we analyzed expression patterns of ten randomly selected probe sets using quantitative RT-PCR (qRT-PCR). Depending on the probe sets analyzed, the correlations between qRT-PCR data and microarray signal intensity varied from 0.531* to 0.932**, indicating that the expression patterns detected by microarray analyses are in good agreement with that detected by qRT-PCR.

#### Hierarchical clustering of HR probe sets

**Figure 2:**
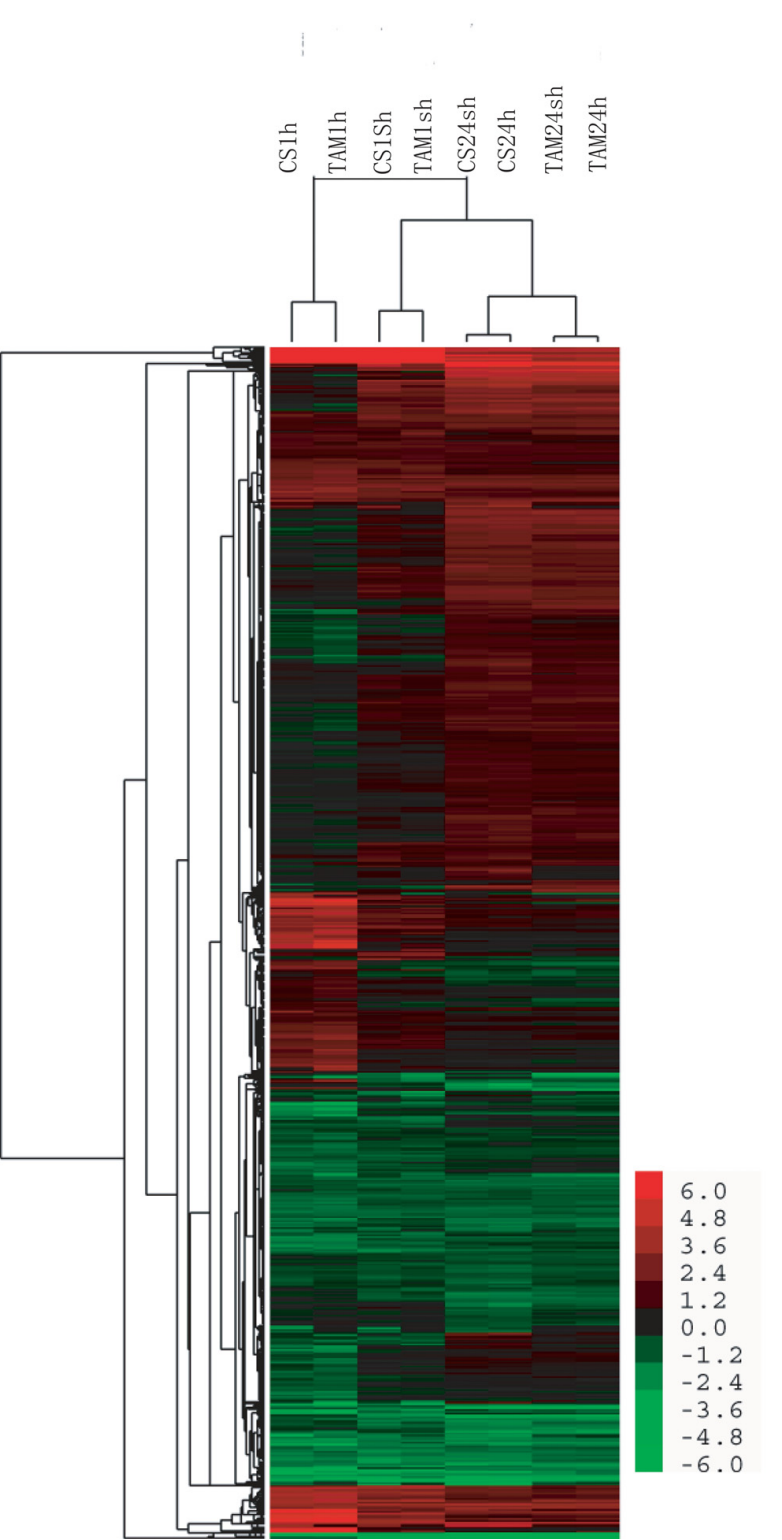
Average linkage hierarchical clustering analysis of the log_2 _transformed changed ratio of the 6560 HR probe sets.

To evaluate the relationship of the genome-wide expression profiles between different genotypes and different treatments, we analyzed linkage hierarchical clustering using 6560 HR probe sets in eight heat-treated samples (Figure 2). The HR genes in the long (24-h) heat treatment were clearly separated from those in the short (1-h) heat treatment. The genes detected under the same short-heat treatment between two genotypes were clustered together (CS1sh with TAM1sh and CS1h with TAM1h), whereas the genes between the two genotypes in the long-heat treatment (CS24sh with CS24h and TAM24sh with TAM24h) were separated, suggesting that gene expression changes under the short heat treatment are dependent on acclimation, and those under the long heat treatment are dependent on genotypes. Cluster analysis also indicated that some genes were up- or down-regulated in all heat treatments tested, while others displayed expression changes in response to different treatments (Figure 2).

#### Classification of HR probe sets based on expression patterns in different genotypes and heat treatments

**Figure 3:**
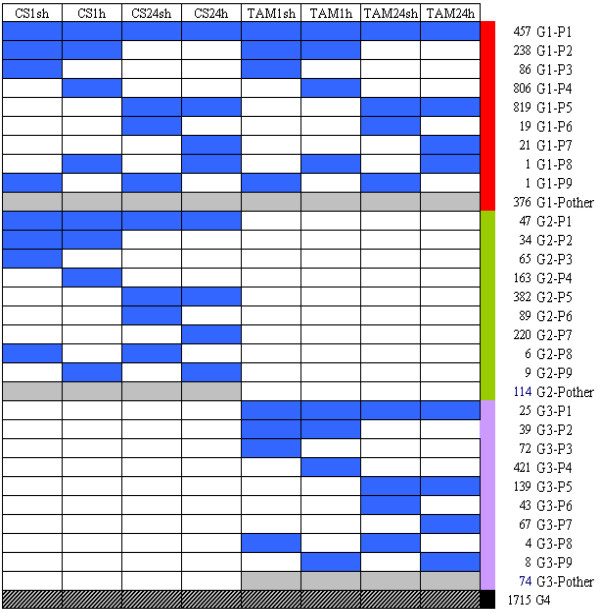
**Classification of expression patterns of 6560 HR probe sets in different genotypes and heat treatments**. The columns represented different heat treatments in two genotypes, and number on the right side of each row represented the numbers of probe sets in each expression pattern. The row marked in gray indicated that the probe sets shown complicated expression patterns.

To identify the co-regulated HR probe sets in different genotypes or heat treatments and genotype/treatment-specific HR probe sets, we classified the 6560 HR probe sets into 4 groups according to their expression patterns in different treatments and genotypes (Figure 3). They were: HR probe sets common in both CS and TAM (Group 1), CS-specific HR probe sets (Group 2), TAM-specific HR probe sets (Group 3), and other HR probe sets (Group 4). The HR probe sets in groups 1–3 were further divided into several subgroups based on different expression patterns (Figure 3, Additional file 3).

**Figure 4:**
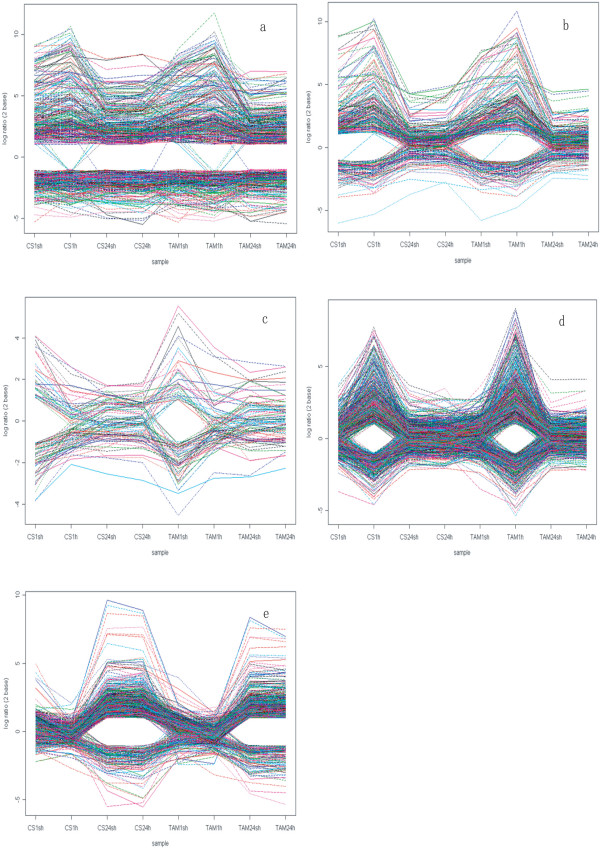
**Expression patterns of probe sets in 5 subgroups of Group1**. The horizontal axes indicated the 8 heat treated samples, and the vertical axes indicated the log2 transformed ratio. (a) G1-P1. (b) G1-P2. (c) G1-P3. (d) G1-P4. (e) G1-P5.

Group 1 contained 2824 (~43% of total HR) probe sets. Group1-Pattern1 (G1-P1) subgroup was heat responsive in all heat treatments (Figure 4a), and 262 and 197 probe sets were up- and down-regulated, respectively. G1-P2 contained 238 probe sets that were responsive only in short heat treatments (1 sh and 1 h) in two genotypes (Figure 4b), and 165 and 72 probe sets were up- and down-regulated, respectively. G1-P3 (86 probe sets) was responsive only to short treatment with pre-acclimation (CS1sh and TAM1sh) (Figure 4c), indicating that they are acclimation-dependent HR genes. In this subgroup, 34 probe sets were up-regulated, and 52 were down-regulated. Among the 806 probe sets that were responsive only to the short treatment without pre-acclimation (G1-P4), 492 were up-regulated, and 314 were down-regulated (Figure 4d), indicating that they are responsive to the heat shock but independent of acclimation. G1-P5 contained 819 probe sets that were responsive to the long heat treatments in both genotypes (Figure 4e), and 705 (86%) probe sets in this subgroup were up-regulated.

A total of 1129 (~17% of the total) HR probe sets were responsive only in 'CS' but not in 'TAM', and therefore designated CS-specific HR probe sets (Figure 3). Among them, nine sub-groups with distinct expression patterns were identified. Expression profiles of the probe sets in subgroups G2-P3, G2-P4, G2-P5, G2-P6 and G2-P7 were shown in Additional file 4. G2-P3 contained 20 up-regulated and 45 down-regulated probe sets that were responsive only in CS1sh, indicating that they were CS-specific and acclimation-dependent genes. G2-P4 contained 163 probe sets that were responsive only to the direct heat treatment without pre-acclimation, and 55 and 108 probe sets were up and down regulated, respectively. A total of 382 probe sets in G2-P5 were responsive in both CS24sh and CS24h, indicating that they were regulated by prolonged heat treatments in 'CS', and 289 were up-regulated, 93 were down-regulated. G2-P6 contained 89 probe sets that were specifically responsive in CS24sh and majority (66) of them were up regulated, indicating that they were responsive to prolonged heat treatment only with pre-acclimation. Moreover, a total of 220 probe sets in G2-P7 were CS24h-specific HR probe sets, either up (158) or down (62) regulated by heat stress.

Group 3 containing 892 (~14% of the total) HR probe sets that were responsive only in 'TAM' but not in 'CS', and therefore designated TAM-specific HR probe sets (Figure 3). The TAM-specific HR probe sets are interesting because 'TAM' is highly tolerant to heat stress. In this group, 9 sub-groups with distinct expression patterns were identified. Subgroup G3-P4 contained 421 probe sets that were responsive only in TAM1h (See Additional file 5), and 179 were up regulated. G3-P5 contained 139 probe sets that were responsive in both TAM24sh and TAM24h, among which 100 were up-regulated (See Additional file 5).

#### Statistical analysis of HR probe sets in different genotypes and heat treatments

Furthermore, we compared the HR probe sets between different genotypes and heat treatments using three factors in the experimental design based on the nested F-test (for detail, see Methods), namely, genotypes (heat susceptible 'CS' and heat tolerant 'TAM'), heat treatments with or without heat acclimation (sh and h), and short or long treatments (1 hour and 24 hours).

**Figure 5:**
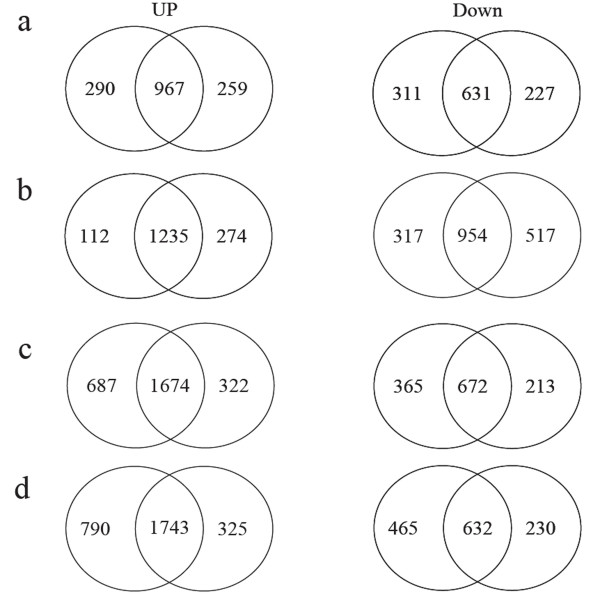
**Overlap of the probe sets up- and down-regulated in 'CS' (left) and 'TAM107' (right) in response to heat stresses. **(a) 1 sh, (b) 1 h, (c) 24 sh, (d) 24 h.

HR probe sets between heat-susceptible 'CS' and heat-tolerant 'TAM': The HR probe sets overlapped substantially between the two genotypes within the same treatment, whereas genotype-specific HR probe sets were also identified (Figure 5). Using nested F-statistic, we found that 47 and 130 probe sets behaved differently between the two genotypes under different heat treatments if the p-value was set to 0.001 and 0.01, respectively. Totally, 313 (P < 0.05) HR probe sets behaved differently between the two genotypes in at least one of the four heat treatments (Table 2), and 18 were in all heat treatments (Table 3), among which 12 and 3 were CS- and TAM-specific, respectively. Among these 313 probe sets, 41 were up- or down regulated in both genotypes. These genes included four HSPs and two splicing coactivator subunit SRm300, which were induced much more in 'CS' than in 'TAM' (Table 3). Except that, they also included protein kinases, protein phosphatase and stress related genes, such as LEAs and peroxidase. For example, phosphatase (PP) 2C was induced for about 4 – fold in CS1h, but for more than 16 – fold in TAM1h, while another protein phosphatase was induced for more than 32 – fold in CS1h, but for 4 – fold in TAM1h. And in *Arabidopsis*, *AtPP7 *has been associated with thermotolerance [17]. Up to 159 probe sets were CS-specific responsive genes, including chlorophyll a/b-binding protein and two oxygen evolving enhancer proteins, and all of them were down regulated by heat stress, suggesting that photosynthesis was damaged more severely in the thermo-sensitive wheat than in the tolerant one. We also found that CS-responsive genes included some biotic stress responsive genes encoding beta-1,3-glucanase precursor, chitinase, PR-1 protein, and leucine-rich repeat domain. A few up-regulated genes encoding transcription factors, such as MYB, MADS, WRKY and also an AN1-like zinc finger, which was induced for more than 32 – fold in CS1h, may contribute to thermo-sensitive phenotype of CS. In contrast, the genes that were specifically responsive in TAM may be of great importance for the good performance of a highly thermo-tolerant genotype under heat stress. The 113 HR genes encoding putative ubiquitin protein, ubiquitin activating enzyme and ribosomal protein were responsive only to TAM1h. The former two types of proteins may help degrade toxic proteins during heat stress, while the latter were responsible for protein biosynthesis, which might help thermotolerant wheat maintain essential metabolism under heat stress. It was intriguing to note that some defense response genes were down-regulated in TAM, including pathogen-induced protein WIR1A, pathogenesis-related protein, stripe rust resistance protein, cell wall invertase, defensin, disease resistance protein, and beta-1,3-glucanase precursor. Actually, the down regulation of genes related to pathogenesis or disease in thermotolerant *Arabidopsis *under heat stress has been reported by other study [18].

**Table 2:** Number of differentially regulated HR probe sets between wheat genotypes 'CS' and 'TAM107' identified by nestF-statistic method.

	1 sh	1 h	24 sh	24 h
Up	3	11	4	2
Down	1	1	4	5
CS-U & TAM-D	0	0	0	0
CS-D & TAM-U	0	0	0	0
CS-U & TAM-NC	20	24	76	81
CS-D & TAM-NC	24	38	25	31
CS-NC & TAM-U	11	28	32	39
CS-NC & TAM-D	12	26	30	31
Total Number	71	128	171	189

*'U' represents up-regulated, 'D' represents down-regulated, and 'NC' represents no change *(FDR p < 0.01).

**Table 3:** Differentially regulated HR probe sets between all heat treatments in 'CS' and 'TAM107'

PID	Annotation	CS1h	CS1sh	CS24h	CS24sh	TAM1h	TAM1sh	TAM24h	TAM24sh
Ta.17450.1.S1_at*	disease resistance protein RPP	0.05	0.00	-0.09	-0.04	-1.86	-1.76	-1.60	-1.73
Ta.22389.1.S1_at*	senescence-associated protein	-0.32	-0.36	-0.28	-0.23	-2.92	-2.80	-4.34	-3.66
Ta.25531.2.A1_x_at*	33 kDa secretory protein	0.19	-0.80	-0.92	-0.86	-1.98	-3.84	-3.88	-3.85
Ta.3464.2.A1_a_at**	MtN21	-2.15	-2.21	-2.23	-2.21	-0.54	-0.57	-0.59	-0.57
Ta.3854.1.S1_at**	MADS-box	2.18	2.68	3.42	3.09	0.29	0.12	0.13	0.19
Ta.774.1.S1_at**	protease	3.49	2.91	2.11	1.93	0.44	0.05	0.02	0.00
Ta.9058.1.S1_at**	beta-fructosidase	-4.25	-4.29	-4.29	-4.17	-0.78	-0.81	-0.65	-0.84
TaAffx.107797.2.S1_s_at**	zinc finger and C2 domain	-1.93	-1.90	-1.84	-1.87	-0.26	-0.31	-0.24	-0.23
TaAffx.131652.1.S1_s_at**	cytochrome P450	-1.21	-1.45	-1.42	-1.38	0.00	-0.01	-0.02	-0.02
TaAffx.29193.1.A1_at**	Protein kinase	-1.74	-1.47	-1.87	-1.68	-0.22	-0.08	-0.21	-0.04
TaAffx.54406.1.S1_at**	disease resistance protein RPM1	-1.85	-2.13	-1.91	-1.87	0.00	-0.04	-0.09	-0.07
TaAffx.71935.1.S1_x_at	splicing coactivator subunit SRm300	5.33	5.12	5.03	4.98	2.70	3.08	3.06	3.31
Ta.11947.1.S1_at**	unknown protein	-1.29	1.31	1.71	1.86	0.01	0.05	0.00	0.00
Ta.12728.3.S1_a_at**	unknown protein	3.16	3.42	3.31	3.44	0.07	0.13	0.09	0.11
Ta.14903.1.S1_at	unknown protein	-3.35	-3.65	-3.68	-3.69	-2.03	-2.00	-2.06	-1.97
Ta.18930.1.A1_at**	unknown protein	-1.17	-1.18	-1.12	-1.13	0.03	0.01	0.00	0.01
Ta.7078.1.S1_at**	unknown protein	-1.75	-1.97	-1.91	-1.86	-0.05	-0.09	-0.10	-0.10
Ta.9179.2.S1_at	unknown protein	1.42	0.52	0.46	0.43	3.28	2.36	2.26	2.03

* TAM107-specific HR probe sets, **CS-specific HR probe sets.

HR probe sets between heat treatments with and without heat acclimation: Heat acclimation under non-lethal high temperature is important for plants to survive at temperature stress that may be lethal [7]. To elucidate the events involved in heat acclimation of wheat, we compared the HR probe sets between the heat-treated samples with and without pre-acclimation. A total of 1314 HR probe sets (See Additional file 6) were differentially regulated between 1 sh and 1 h heat treatments based on nest-F test (p < 0.001), and no probe sets were found to be significantly differentially regulated between 24 sh and 24 h treatments either in magnitude or in direction (Table 4), suggesting that heat acclimation had no effect on expression during the prolonged heat treatment. We focused on the 823 probe sets that were differentially regulated between 1 sh and 1 h both in CS and TAM, considering that they were real difference between treatment with and without pre-heat acclimation. They were further classified into the following functional categories: (1) transcription factors, including C2H2 zinc finger, MYB, HSF, bZIP, NAC, AP2, bHLH and WRKY; (2) RNA metabolism, including RNA polymerase, mRNA capping enzyme, double-stranded RNA binding motif, RNA helicase, RNA recognition motif, RNA processing factor, U3 snoRNP protein, U2 snRNP auxiliary factor, nuclear RNA binding protein and valyl-tRNA synthetase; (3) protein biosynthesis and degradation, including ubiquitin/ribosomal polyprotein, ubiquitin carboxyl-terminal hydrolase, ubiquitin conjugation factor, ribosomal protein and polyubiquitin; and (4) signal transduction, including calcium binding protein, PP2C, protein kinases. The results suggest that heat acclimation affects the expression of genes involved in transcriptional regulation as well as metabolic pathways.

**Table 4:** Differentially regulated HR probe sets between heat treatments with and without heat acclimation identified by nestF-statistic method.

	CS1sh vs CS1h	CS24sh vs CS24h	TAM1sh vs TAM1h	TAM24sh vs TAM24h
Up	87	0	113	0
Down	8	0	12	0
sh-U & h-D	14	0	25	0
sh-D & h-U	3	0	21	0
sh-U & h-NC	237	0	240	0
sh-D & h-NC	33	0	39	0
sh-NC & h-U	336	0	459	0
sh-NC & h-D	131	0	247	0
Total Number	849	0	1156	0

*'U' represents up-regulation, 'D' represents down-regulation, NC' represents no change *(FDR p < 0.001).

HR probe sets between short (1 hour) and prolonged (24 hours) heat treatment: We also compared the expression profiles between 1-hour and 24-hour heat treatments (See Additional file 7) to identify the genes involved in short and prolonged heat treatments. Nested F-statistic analysis showed that 4533 probe sets (p < 0.001) were differentially regulated between the two treatments (See Additional file 8), accounting for 69% of total HR probe sets. Among these probe sets, 1355 were between CS1sh and CS24sh, and 1278 were between TAM1sh and TAM1h. However, much more probe sets were differentially regulated between 1 h and 24 h, 3426 and 3405 were identified in CS and TAM, respectively. Considering the large number of probe sets, we focused on the 966 probe sets that were differentially regulated between all the four pair-wise comparisons. The genes encoding heat shock proteins and ribosomal proteins were among those genes. Moreover, these probe sets also included genes encoding putative zinc-finger protein, protein phosphatase and kinase, oxidative stress responsive genes (sulfur-rich/thionin-like protein, ferritin, GST, peroxidase), metabolism (GTP-binding protein, chlorophyll a/b-binding protein).

### Major functional categories of the HR genes in wheat

A large number of HR genes do not match any genes with know functions probably because the wheat genomic information is very limited. Our further analysis was focused on the HR probe sets that have been assigned to annotated genes and ESTs by HarvEST [19], which account for ~75% of the HR probe sets.

#### Heat shock proteins (HSPs) and other chaperones were highly induced by heat stress

**Figure 6:**
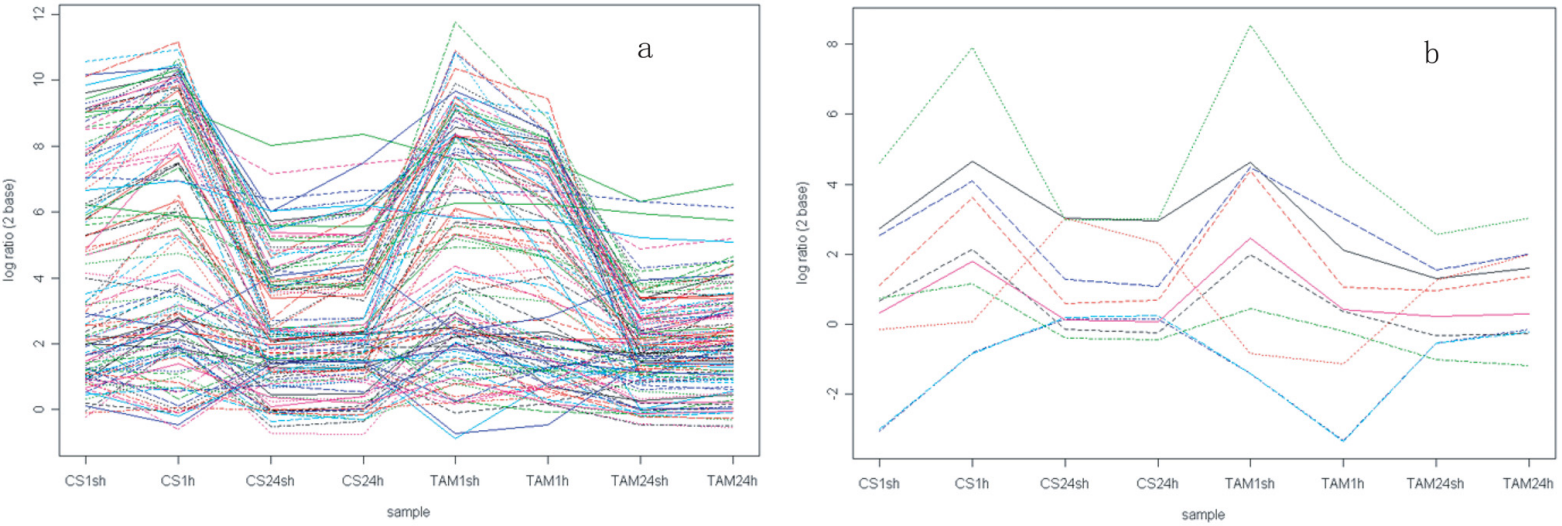
**Expression patterns of probe sets representing genes encoding heat shock proteins and heat shock factors**. (a) Heat shock proteins. (b) Heat shock factors.

The induction of heat shock proteins when plants are exposed to elevated temperature has been well documented [20-22]. HSPs function as molecular chaperones in maintaining homeostasis of protein folding and are related to the acquisition of thermotolerance [23]. Coincident with this, 117 probe sets encoding various HSPs were up-regulated by the heat treatments in wheat, and the highest fold change was 11.8 (Log 2 transformed, Figure 6a). We also observed that expression of these HSPs altered more in 1-h heat treatment than in 24-h heat treatment. Seven HSFs were induced by heat stress (Figure 6b), and one (Ta.28772.1.S1_at) was induced for more than 128 – fold in 1 h. However, two probe sets representing HSFs were down-regulated after heat stress. This was inconsistent with studies in *Arabidopsis*, in which all the six heat responsive HSFs were up-regulated by the 1-hour heat treatment [16]. Peptidyl prolyl cis-trans isomerase is another chaperone family that has been reported to be induced by heat in wheat [24] and *OsFKBP20 *over-expression in yeast endowed capacity of high temperature tolerance in yeast cells [25]. In this analysis, 18 probe sets encoding peptidyl prolyl cis-trans isomerase were up-regulated by heat treatment, among which one (Ta.639.1.S1_at) was elevated for more than 26-fold in all heat treatments.

#### Transcription Factors (TFs) responsive to heat stress

In addition to HSFs, *DREB2A *was reported to regulate heat tolerance [26] and act as a transcriptional activator of *HsfA3 *[27]. In our study, two probe sets encoding *DREB2B *and *DREB6A *were up-regulated by heat stress. Another three probe sets annotated as *ethylene-responsive transcriptional coactivator *(*ERETC*) gene, a member of *MBF1 *(Multiprotein bridging factor), were up-regulated more than 8-fold in all heat treated samples. Previous study showed that *AtMBF1c *was strongly induced by both heat stress [12] and a combination of drought and heat stresses [10], and over-expression of the *AtMBF1c *enhanced the tolerance to both heat and osmotic stresses of *Arabidopsis *[28]. The strong induction of *ERETC *expression by heat stress in wheat provided another piece of evidences for its role in thermotolerance.

**Figure 7:**
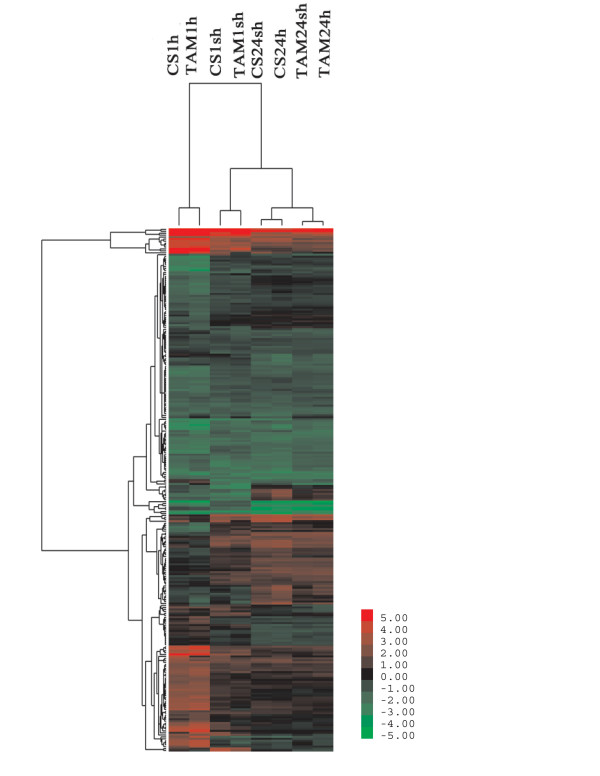
Average linkage hierarchical clustering analysis for probe sets representing genes encoding transcription factors.

Many other transcription factor (TF) genes were also affected by heat stress in wheat, although their roles in heat tolerance are not clear. These TF genes included 162 probe sets for zinc-fingers genes, 53 for MYB genes, 11 for AP2 genes, 24 for bZIP genes, 8 for bHLH genes, 8 for NAC or NAM genes, 11 for WRKY genes, and genes for other factors, such as GRAS, MADS box and auxin response factor (ARF). The expression patterns of these HR TF genes were complex in various heat treatments (Table 5). Interestingly, the majority of HR TF genes were responsive in the heat shock treatment without pre-acclimation (1 h), and most of them were down-regulated (Table 5, Figure 7). The data were in agreement with the previous report in *Arabidopsis *[16]. *AN1-like *(Ta.5930.1.S1_s_at) and *C3HC4-type RING fingers *(TaAffx.20445.1.S1_at) genes were strongly up-regulated after 1 h treatment, and *C3HC4-type finger *(TaAffx.20445.1.S1_at) gene was up-regulated 16-fold in both genotypes. One *NAC *(Ta.16423.1.S1_at) gene was down-regulated 16-fold in 1 h, 24 sh and 24 h treatments in two genotypes with a less extent in 1 sh treatment. Two probe sets encoding putative WRKY factors were induced more in 1 h than in 1 sh in both genotypes. Intriguingly, one probe set (Ta.4725.1.S1_at) was repressed in TAM1sh but was induced in TAM1h, which may suggest different roles of this gene in acquired and basal thermotolerance. The function of *WRKY *genes in plant defense responses has been well characterized [29], and the expression of some members of this gene family was also affected by high temperature [30].

**Table 5:** Numbers of transcription factors up- and down-regulated in different heat treated samples.

Transcription factors	No. of HR probe sets*	CS1sh	CS1h	CS24sh	CS24h	TAM1sh	TAM1h	TAM24sh	TAM24h
Heat shock factor	11	2+,2-	6+,0-	2+,0-	2+,0-	2+,2-	6+,0-	1+,1-	3+,1-
zinc finger	162	42+,33-	48+,40-	34+,43-	34+,48-	41+,30-	53+,49-	27+,37-	31+,39-
MYB	53	0+,17-	5+,27-	1+,20-	4+,25-	2+,18-	7+,30-	2+,15-	4+,17-
AP2	11	3+,2-	2+,3-	1+,3-	2+,3-	3+,1-	3+,4-	1+,2-	2+,1-
bZIP	24	3+,3-	6+,9-	1+,5-	1+,4-	4+,1-	5+,6-	4+,7-	2+,4-
bHLH	8	0+,1-	1+,1-	0+,0-	0+,0-	0+,1-	5+,0-	0+,1-	0+,1-
NAC and NAM	8	0+,2-	4+,3-	0+,3-	0+,4-	0+,2-	4+,3-	0+,3-	0+,3-
WRKY	11	3+,0-	5+,0-	2+,0-	3+,2-	3+,1-	7+,0-	1+,0-	2+,0-

* Total number of HR probe sets, +, up-regulated, -, down-regulated.

#### Phytohormones and wheat heat response

The roles of phytohormones, including abscisic acid (ABA), brassinosteroids (BRs) and ethylene, in heat tolerance have been well documented [20,31-33]. We found that the candidate genes involved in phytohormone metabolism or signaling, including ABA, auxin, ethylene and gibberellic acid (GA), were regulated by heat stress (See Additional file 9).

*ABA-INSENSITIVE2*, a component of ABA signaling pathway was up-regulated 7- and 28-fold, respectively, in CS1h and TAM1h. *AREB2 *and *AREB3*, also involved in ABA signaling, were down-regulated by the same heat treatments. Three putative homologs of Arabidopsis *Arac7 *and *Arac10*, the negative regulator of ABA-mediated signaling [34], were down-regulated by all heat treatments. Four probe sets encoding ABA-induced *HVA22 *were up-regulated by the heat treatments especially in the 1 h treatment. These data indicated that ABA signaling is involved in the heat shock response in wheat. Additional experiments are needed to determine their roles and downstream pathways in thermotolerance.

A link between ethylene production and heat susceptibility in wheat was reported recently [35]. In our study, we identified several HR genes in ethylene biosynthesis/signaling, including one for *ACC synthase*, seven for *ACC oxidases*, 12 for MAPKs and two for CTR1-like kinases. Six probe sets encoding *ACC oxidases *were down-regulated by heat stress, and one probe set for ACC synthase was also down-regulated by all the heat treatments, suggesting a negative response to ethylene level during heat stress. The candidate genes encoding MAPKs and a CTR1 kinase involved in the ethylene signal pathway were also repressed by 1-h or 24-h heat treatment. Candidate genes responsive to ethylene biosynthesis, such as *ERETC *just mentioned, *chitinase *and *ER*s (ethylene responsive genes), were also among the HR probe sets. A *endochitinase-like protein *gene was shown to be essential for heat, salt and drought tolerance [36].

The F-box-containing auxin receptors (*TIR1*) accelerate the 26S proteasome-dependent degradation of short-lived transcriptional repressors, AUX/IAA, which in turn allows ARFs to modulate gene expression by binding to the promoters of auxin regulated genes [37-39]. In this analysis, seven probe sets encoding AUX/IAA proteins were down-regulated by heat stress, whereas three putative HR genes encoding 26S proteasome regulatory subunits were up-regulated. Two genes encoding putative *TIR1*-like proteins, *ARF3 *and *ARF10*, were heat induced, and *ARF10 *(TaAffx.34454.1.S1_at) was induced for more than 8-fold in 1 sh and 1 h treatments in both genotypes. This implied that a positive response of auxin level to heat stress. Alternatively, high temperature stress may activate auxin signal pathway. Moreover, six probe sets encoding putative expansin were induced by the long heat treatment. The expression level of expansin gene *AsEXP1 *in different *A. scabra *genotypes was positively correlated with the level of heat tolerance in this grass species [40], which is reminiscent of the up-regulation of putative expansin genes in heat tolerant TAM compared to heat susceptible CS.

The role of gibberellic acid (GA) in plant thermotolerance is still debatable [41,42] and GA was thought to play a role in antioxidant pathway [43,44]. We found that the genes involved in GA biosynthesis, including *GA3 *and *2OG-Fe oxygenase*, were responsive to heat stress (Additional file 9). *RGA*, the negative regulator of GA signaling, is a member of DELLA protein [45]. A putative wheat *RGA *was down-regulated by heat stress, and Ta.11162.1.S1_at encoding putative GA regulated protein *GAST1 *precursor was strongly (log2 > 5.1) induced in all eight heat treatments, suggesting a role in heat response in wheat. Indeed, some members of *GASA *family in *Arabidopsis *are associated with heat stress response [22], and over-expression of *GASA *enhanced the thermotolerance [46].

#### Calcium signaling

Calcium is a universal signaling molecule in both animals and plants, and the transient increase of Ca^2+ ^level during heat stress was well documented in plants [47]. Heat shock triggers cytosolic Ca^2+ ^bursts, which is transduced by Ca^2+ ^binding proteins (CBP) such as calmodulin (CaM), CaM-related proteins, Ca^2+^-dependent protein kinases (CDPK), and calcineurin B-like protein (CBL), and then up-regulates the expression of HSPs [47,48]. In this analysis, candidate genes encoding the components of calcium or calmodulin mediated signal pathway, including *annexin*, *CBPs*, *CDPKs*, *voltage-gated calcium channel activity*, Ca2+-binding protein EF hand, *CBL *and *CIPK *(*CBL-interacting protein kinase*), were also heat regulated, suggesting a role of Ca^2+ ^mediated signal in wheat heat stress response. Indeed, some of these genes, such as *annexin1 *[49] and *CDPK *[13], are induced by heat stress.

#### Sugar signaling

The metabolomic analysis under high temperature stress indicated that sugar content increased very rapidly and maintained throughout heat treatments [50]. Parallel microarray analysis revealed that the promoters of a number of genes induced by heat shock contained sugar-responsive elements [50], suggesting that sugar signaling is important in the establishment and maintenance of acquired thermotolerance. In wheat, the genes in sugar-mediated signaling were regulated by heat stress. For example, four probe sets encoding the homolog of replication licensing factor *MCM7 *were induced in 1 h, and one (TaAffx.32695.1.S1_s_at) of them was induced more than 64-fold in CS1h and TAM1h. Five probe sets for *glucose-6-phosphate/phosphate-translocator *were also regulated by heat stress, and one of them was up-regulated 8-fold or more in CS1h and TAM1h. Eight probe sets for *fructokinase*, related to sugar sensing in plants [51], were also heat regulated, and five of them were up-regulated. We also found that 22 probe sets for sugar transporters were responsive to heat stress, and three of them were up-regulated.

#### RNA metabolism

Previous study demonstrated that alternative splicing of pre-mRNAs of Arabidopsis serine/arginine-rich proteins was regulated by heat stress [52]. A glycine rich-RNA binding protein from rice is responsive to high temperature stress, and its over-expression disrupts thermotolerance in wild type yeast cells [53]. Two genes encoding Arabidopsis DEAD-box RNA helicases, *STRS1 *and *STRS2*, are the negative regulators of heat tolerance [54]. Expression of 239 probe sets for the genes in RNA metabolism was affected by heat stress in wheat, and nearly half of them encode putative RNA binding proteins, including 12 probe sets for glycine-rich RNA binding proteins, 14 for ribonucleoproteins, and 40 for RNA recognition motif (RRM)-containing proteins. Some probe sets represented the genes related to RNA processing or modification, including RNA polymerase, mRNA capping enzyme, pre-mRNA splicing factor, RNA helicase, RNA methyltransferase, and U2 snRNP auxiliary factor. The genes encoding putative ASF/SF2-like pre-mRNA splicing factors and splicing coactivator subunit SRm300 were strongly induced by all heat treatments. Specifically, 43 HR probe sets were involved in tRNA synthesis, such as aspartyl-tRNA synthetase, trytophanyl-tRNA synthetase and phenylalanyl-tRNA synthetase. tRNAs are responsible for the transportation of amino acid during protein biosynthesis. We found that the expression levels of 41 probe sets for tRNAs were increased during 24-h heat treatments, suggesting an increased level of protein biosynthesis during the long-term heat stress. Significantly, several genes involved in the biogenesis of siRNA and miRNA, including *Dicer *homolog, *AGO1*, *piwi*, and dsRNA-binding proteins, were also regulated by heat stress, Several microRNA (miRNA) target genes, such as *SPL2 *(miR156) [55] and *ARF16 *(miR160) [56], were down-regulated, suggesting a role of miRNAs in heat response pathway in plants.

#### Ribosomal proteins

**Figure 8:**
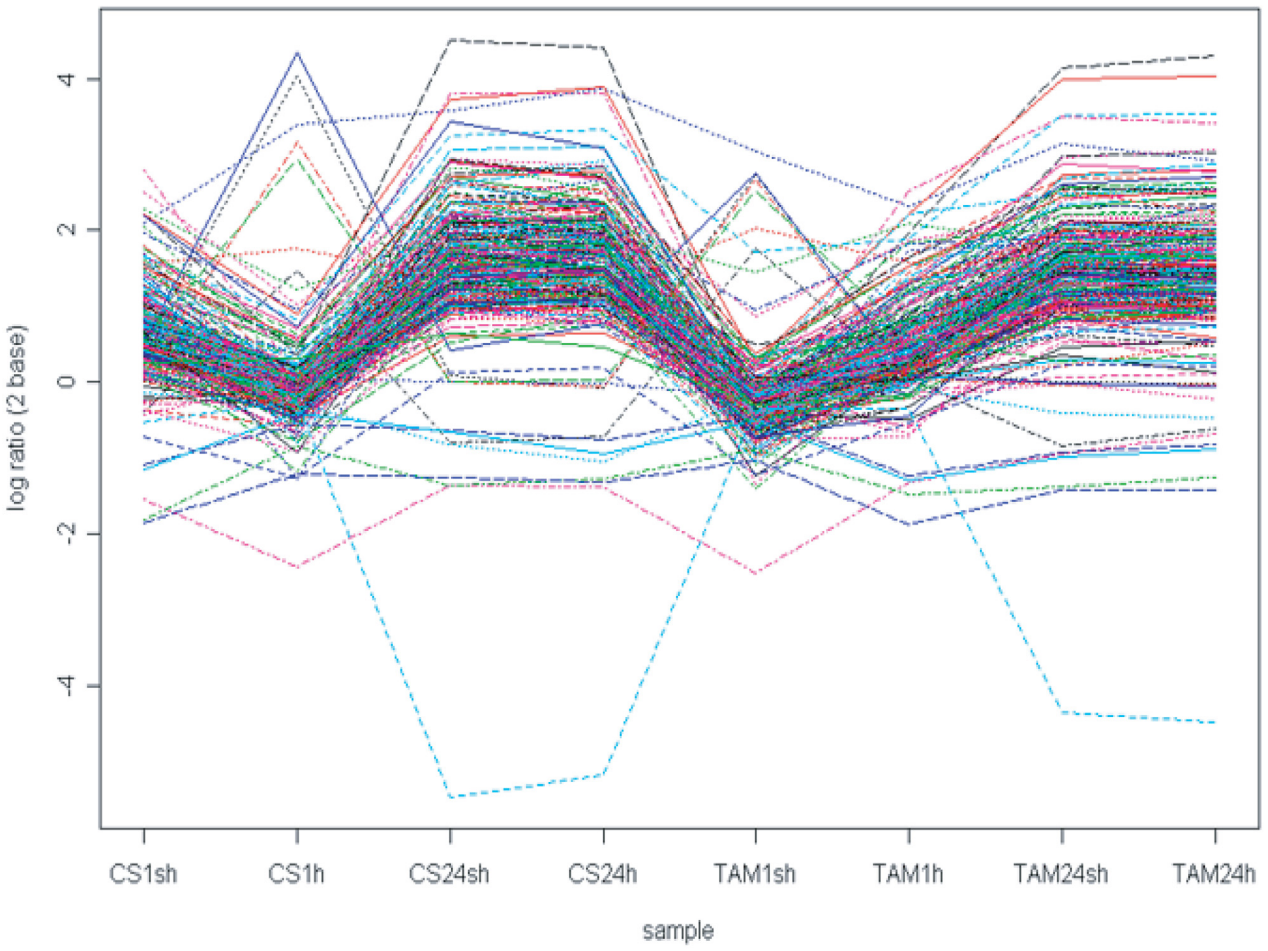
Expression pattern of probe sets representing genes encoding ribosomal proteins.

Ribosomal proteins play an important role in escaping protein synthesis from being inhibited by heat stress [57,58]. We found that 264 probe sets for ribosomal protein were up-regulated under the long-term heat treatment (Figure 8). Notably, other components of the translation system, such as the genes encoding translation initiation factors and translation elongation factors, were also induced in the late time of heat treatments, indicating that protein synthesis is responsive to heat stress, especially in the long-term heat conditions.

#### Extensive remodeling of metabolism under heat stress

Metabolites can act as signaling/regulatory agents, compatible solutes, and antioxidants or in defense against pathogens [50]. Temperature is one of the most active environmental factors that affect all plant metabolic activities, including amino acid and carbohydrate metabolism [50]. Secondary metabolites is involved in resistance against heat shock [59]. We found that the genes functioning in primary or secondary metabolisms were affected by heat stress.

Photosynthesis is one of the most heat-sensitive processes. The damage of photosystem II (PSII) and Rubisco activity by high temperature is irreversible [11]. We found that 21 probe sets for chlorophyll a/b-binding protein were repressed by heat stress especially in the 24-hour heat treatments, which suggesting that long-term heat stress damaged the photosystems.

The gene shuffling technology has been applied to generate several *Arabidopsis thaliana RCA1 *(Rubisco Activase 1) variants, and the transgenic lines with the thermostable *RCA1 *variants exhibited higher photosynthetic rates, improved development patterns, higher biomass, and increased seed yields [60]. Under heat stress, we found that the RuBisCO activase B gene was induced more in TAM than in CS. The genes encoding PSI reaction center subunits were also repressed by heat stress, especially in CS24h, suggesting that a severe damage is caused by high temperature in the thermo-susceptible wheat. Other genes responsible for photosynthesis, such as oxygen evolving enhancer protein were also down-regulated by heat stress. In addition, plant respiration was also a heat sensitive physiological process. We found that 40 genes encoding electron transporters, including cytochrome b5, Glutaredoxin, disulfide-isomerase and rubredoxin, were affected by heat stress. Cytochrome *P450s *have been suggested to play roles in protecting organisms from oxidative damage, and the induction of *P450 *is related to different biotic and abiotic stresses [61]. In this study, 30 probe sets for *P450 *were heat responsive, and they may play a role in the detoxification during heat stress.

Several probe sets representing genes related to carbohydrate metabolism were regulated by heat stress in our experiment, including sugar and trehalose metabolism, such as sugar transporters, sucrose transporters, trehalose-6-phosphate phosphatase (*TPP*), trehalose-phosphate synthase (*TPS*), and cell wall invertase. Trehalose not only acts as a carbohydrate reserve but also plays an important role in plant abiotic stress tolerance [62]. TPP and TPS are known to be the key enzymes in the biosynthesis of trehalose [63-65]. Indeed, *TPP *is induced by heat stress, and *TPP *complements the default thermotolerance of *TPS *yeast mutant, a *TPP *homolog in other organisms [66].

Lipid metabolism is also heat regulated in this study. We found that 22 probe sets encoding lipid transfer proteins were regulated by long-term heat stress. In *Arabidopsis*, *fad7fad8 *double mutant was resistant to high temperature [67]. In our study, a wheat *FAD3 *was down-regulated in CS1sh and TAM1sh, indicating a role of pre-acclimation in heat stress probably through an increase in the fluidity of cell membrane under high temperature.

#### Genes related to other abiotic stresses are also regulated by heat stress

Heat stress can induce oxidative stress through the formation of free radicals [50] and reactive oxygen species (ROS). There is inter-linking between heat and oxidative stress responses [33]. Arabidopsis lacking *thylakoid ascorbate peroxidase *(*tylapx*) has enhanced basal thermotolerance [68]. We found that the HR genes include those encoding putative NADPH oxidase, mitochondrial alternative oxidase (AOX), germin-like oxalate oxidases and amine oxidases, which are related to ROS production [69]. The genes encoding enzymatic components of ROS-scavenging pathways, including ascorbate peroxidase, superoxide dismutase, glutathione-S-transferase (GST), glutathione peroxidase, ferritin and thioredoxin [69], were also affected by heat stress, suggesting that a link between these two stress signaling pathways. Two of four putative NADPH oxidase genes were induced by 24-h heat stress, but the other two were repressed by 1-h treatments, suggesting induction of oxidative stress by long-term heat treatments. Most of the genes involved in ROS-scavenging pathway, including *AOXs*, *APX3*, *glutathione peroxidases*, *thioredoxin*, *ferritin *and *thioredoxin*, showed higher expression levels during the short-term heat shock than the long-term heat treatments. The data suggest that plants can scavenge the ROS generated by heat shock, but the oxidative stress induced by long-term heat stress may impair their ability to generate these beneficial molecules.

The effects of drought and heat stress on cereals are interlinked [70]. We found that some HR probe sets were assigned to be responsive to drought or desiccation. The genes encoding putative osmoprotectants, such as mannitol, trehalose and late embryogenesis abundant (LEA) proteins, were affected by high temperature, suggesting a common mechanism for heat, drought and other osmotic stresses. Moreover, some major intrinsic protein (*MIP*), tonoplast intrinsic protein (*TIP*) and ion transporters may also play an important role because some HR probe sets for these proteins were affected by heat stress.

## Conclusion

Using Affymetrix Wheat Genome Array, we found that ~11% (6560) of total probe sets were responsive to short and prolonged heat stress treatments in wheat. First, the two wheat genotypes with contrasting heat stress responses displayed different gene expression changes in short and prolonged heat treatments. Second, heat acclimation had minor effects on gene expression in the prolonged heat treatment but had measurable effects on gene expression in the short-term heat stress. Third, different genes and pathways were up- or down-regulated under the short and prolonged heat treatments, and more genes in metabolism and protein synthesis were affected by the prolonged heat treatment than the heat shock. Fourth, the heat responsive genes identified in this study belong to a large number of important factors and biological pathways, including HSPs, transcription factors, phytohormone biosynthesis/signaling, calcium and sugar signal pathways, RNA metabolism, ribosomal proteins, primary and secondary metabolisms, and biotic and abiotic stresses. We have identified several sets of common genes and factors as well as novel genes responsive to heat stress, which may help elucidate molecular bases for thermotolerance and heat tolerance in wheat and other plant species.

## Methods

### Plant materials and heat treatments

Two wheat genotypes, heat susceptible 'Chinese Spring' (CS) and heat tolerant 'TAM107' were used in this study. Seeds were surface-sterilized in 1% sodium hypochlorite for 15 min, rinsed in distilled water, and soaked in dark overnight at room temperature. The germinated seeds were transferred into the pots (25 seedlings per pot) containing vermiculite. A total of 75 seedlings in three pots were used for each treatment, and three independent biological replications were employed. Prior to heat treatments, the seedlings were grown for 10 days in a growth chamber with 22°C/18°C (day/night), 12 h/12 h (light/dark), and 60% humidity. All heat treatments were carried out at the beginning of the light cycle to avoid circadian rhythm variation [71]. The seedlings that were subjected to 40°C (heat stress) for 1 hour with and without heat acclimation (34°C, 3 hours) designated 1 sh and 1 h, respectively, and the seedlings that were subjected to 40°C for 24 hours with and without heat acclimation (34°C, 3 hours) designated 24 sh and 24 h, respectively (Figure 1). At the end of heat treatments, the leaves of the seedlings from the three pots were pooled together as one biological replicate and frozen immediately in the liquid nitrogen, and stored at -80°C for further use. A total of 30 samples including controls were prepared from three independent biological replications.

### Microarray analysis

Total RNA was extracted using TRIzol reagent (Invitrogen) following the manufacturer's recommendations. Briefly, mRNA was enriched from 80~90 μg total RNA using the RNeasy Plant Mini Kit (QIAGEN) according to the instructions and was subsequently reverse-transcribed to double stranded cDNA using the GeneChip^® ^Two-Cycle cDNA Synthesis Kit. The biotin-labeled cRNA was made using the GeneChip^® ^IVT Labelling Kit (Affymetrix, CA, USA). Twenty micrograms of cRNA samples were fragmented and hybridized for 16 hours at 45°C to the Affymetrix Wheat Genome Array (Santa Clara, CA, USA). After washing using the Genechip^® ^Fluidics Station 450, the arrays were scanned using the Genechip^® ^3000 Scanner that is located in the Bioinformatics Center at China Agricultural University. The microarray data for this paper have been deposited in the public repository of ArrayExpress [72] (ArrayExpress accession Number: E-MEXP-1523).

### Data analysis

The chip images were scanned and extracted using default settings and the CEL files were produced with the Affymetrix GeneChip Operating Software (GCOS 1.2). The resulting CEL files were imported into the Bioconductor (version 2.0) [73] in R 2.5.1 statistical environment. Subsequent background adjustment, normalization of the raw data and estimation of probe sets signal intensities were done using GC-RMA method [74]. In order to assess the reproducibility of the experiment, the normalized signal intensities from 3 replications of each sample were used to calculate correlation coefficients.

To identify HR probe sets, the probe sets prefixed with "AFFX" and "RPTR" were removed and filtered by fraction call 100% suggested by McClintick and Edenberg [75]. A total of 26,669 probe sets that were 'present' in at least one sample were selected for analysis. Fold changes and p-values of probe sets were calculated using limma [76] nested F-test, and the p-values for multiple testing were corrected using the false discovery rate (FDR) [77]. As a result, 6560 were identified as HR probe sets (cut-off: adjusted p-value = 0.001, log_2_FoldChange = 1).

The identification of HR probe sets that were significantly different in expression between two of the three factors (genotypes, heat treatments with and without acclimation, 1-hour and 24-hour heat treatments) was performed using the same method as used in the identification of HR probe sets. The fold change was 2 or larger, and the FDR adjusted p-values were 0.05, 0.001, and 0.001, respectively.

Annotations of the wheat probe sets were obtained from HarvEST: Affymetrix Wheat 1 Array 1.12 [19]. Cluster software [78] was used to perform hierarchical clustering analysis using Euclidean Distance and average linkage method to create probe sets and sample trees. JAVA Treeview [79] was used to view cluster image.

### Quantitative Real-Time PCR (QRT-PCR) Analysis

A portion of the seedling leaves that were used for microarray analysis was used for qRT-PCR analysis to validate the expression patterns. Total RNA was extracted using TRIzol reagent (Invitrogen). Two microgram total RNA was used to synthesize first-strand cDNA using oligo (dT)_18 _primer with M-MLV reverse transcriptase (Promega, USA) according to the manufacturer's instructions. The product of reverse transcription was tested by amplifying a wheat actin gene fragment (330-bp), which was used as the endogenous control. PCR primers were designed using DNAMAN software, and the primer pairs used to amplify probe set-specific products were listed in Additional file 10. QRT-PCR was performed using the cDNA samples in a 10-μL mixture containing 1 × LightCycler-FastStart DNA master SYBR Green I. PCR was performed as follows: initial denaturation for 10 min at 95°C, followed by 40 cycles of 30 s at 95°C, 45 s at 55 to 60°C, 10 s at 72°C with a final extension at 72°C for 5 min. The threshold cycles (Ct) of each test target were averaged for triplicate reactions, and the values were normalized according to the Ct of the control products (*Ta-actin*). Each PCR product was evaluated in at least two independent experiments.

## Authors' contributions

DDQ performed the microarray experiments and drafted the manuscript, HYW performed computational analysis of the array data, HRP and CLZ grew wheat plants and performed heat stress experiments, ZXL performed qRT-PCR assays, ZFN and YYY analyzed the data, and QXS designed the experiments and revised the manuscript. All authors have read and approved the final manuscript.

## Supplementary Material

Additional file 1 **Correlation coefficient of any two of the three replications of the 10 samples.** Click here for file

Additional file 2 **Log2 values of the 6560 HR probe sets in different heat treatment as compared to the untreated control samples.** Click here for file

Additional file 3 **Classification of HR probe sets based on expression patterns in different genotypes and heat treatments.** Click here for file

Additional file 4 **Expression patterns of the probe sets in Group 2**. (a) G2-P3, (b)G2-P4, (c) G2-P5, (d) G2-p6, (e) G2-P7. Click here for file

Additional file 5 **Expression patterns of probe sets in Group 3**. (a) G3-P4, (b) G3-P5. Click here for file

Additional file 6 **Overlap of the probe sets regulated in response to heat treatments with and without pre-acclimation**. (a) CS1sh (left) and CS1h (right), (b) CS24sh and CS24h, (c) TAM1sh and TAM1h, (d) TAM24sh and TAM24h. Click here for file

Additional file 7 **Overlap of the probe sets regulated in response to 1-h and 24-h heat treatments**. (a) CS1sh and CS24sh, (b) CS1h and CS24h, (c) TAM1sh and TAM1h, (d) TAM1h and TAM24h. Click here for file

Additional file 8 ** Differentially regulated HR probe sets between 1 hour and 24 hours heat treatments identified by nested F-statistic method**. 'U' represents up-regulation, 'D' represents down-regulation, 'NC' represents no change (FDR p < 0.001). Click here for file

Additional file 9 **Expression patterns of HR probe sets representing genes in hormone biosynthesis and signaling pathway.** Click here for file

Additional file 10 **Primer pairs for qRT-PCR.** Click here for file
