# Supplementary material for: Heat stress-responsive transcriptome analysis in heat susceptible and tolerant wheat (Triticum aestivum L.) by using Wheat Genome Array
Source: BMC Genomics. 2008 Sep 22;9:432. doi: 10.1186/1471-2164-9-432 (PMC2614437; doi:10.1186/1471-2164-9-432)
Supplement: Additional file 1 — Correlation coefficient of any two of the three replications of the 10 samples. [file 1471-2164-9-432-S1.doc]

***Additional file 1: Correlation coefficients of any two of the three replications of the 10 samples***

|  | CSck-1 | CSck-2 | CSck-3 |  |  | TAMck-1 | TAMck-2 | TAMck-3 |
| --- | --- | --- | --- | --- | --- | --- | --- | --- |
| CSck-1 | 1.000 | 0.988 | 0.972 |  | TAMck-1 | 1.000 | 0.989 | 0.965 |
| CSck-2 | 0.988 | 1.000 | 0.980 |  | TAMck-2 | 0.989 | 1.000 | 0.969 |
| CSck-3 | 0.972 | 0.980 | 1.000 |  | TAMck-3 | 0.965 | 0.969 | 1.000 |
|  |  |  |  |  |  |  |  |  |
|  | CS1h-1 | CS1h-2 | CS1h-3 |  |  | TAM1h-1 | TAM1h-2 | TAM1h-3 |
| CS1h-1 | 1.000 | 0.982 | 0.956 |  | TAM1h-1 | 1.000 | 0.983 | 0.976 |
| CS1h-2 | 0.982 | 1.000 | 0.957 |  | TAM1h-2 | 0.983 | 1.000 | 0.968 |
| CS1h-3 | 0.956 | 0.957 | 1.000 |  | TAM1h-3 | 0.976 | 0.968 | 1.000 |
|  |  |  |  |  |  |  |  |  |
|  | CS1sh-1 | CS1sh-2 | CS1sh-3 |  |  | TAM1sh-1 | TAM1sh-2 | TAM1sh-3 |
| CS1sh-1 | 1.000 | 0.981 | 0.972 |  | TAM1sh-1 | 1.000 | 0.987 | 0.983 |
| CS1sh-2 | 0.981 | 1.000 | 0.977 |  | TAM1sh-2 | 0.987 | 1.000 | 0.982 |
| CS1sh-3 | 0.972 | 0.977 | 1.000 |  | TAM1sh-3 | 0.983 | 0.982 | 1.000 |
|  |  |  |  |  |  |  |  |  |
|  | CS24h-1 | CS24h-2 | CS24h-3 |  |  | TAM24h-1 | TAM24h-2 | TAM24h-3 |
| CS24h-1 | 1.000 | 0.987 | 0.989 |  | TAM24h-1 | 1.000 | 0.992 | 0.981 |
| CS24h-2 | 0.987 | 1.000 | 0.980 |  | TAM24h-2 | 0.992 | 1.000 | 0.979 |
| CS24h-3 | 0.989 | 0.980 | 1.000 |  | TAM24h-3 | 0.981 | 0.979 | 1.000 |
|  |  |  |  |  |  |  |  |  |
|  | CS24sh-1 | CS24sh-2 | CS24sh-3 |  |  | TAM24sh-1 | TAM24sh-2 | TAM24sh-3 |
| CS24sh-1 | 1.000 | 0.991 | 0.986 |  | TAM24sh-1 | 1.000 | 0.994 | 0.983 |
| CS24sh-2 | 0.991 | 1.000 | 0.983 |  | TAM24sh-2 | 0.994 | 1.000 | 0.983 |
| CS24sh-3 | 0.986 | 0.983 | 1.000 |  | TAM24sh-3 | 0.983 | 0.983 | 1.000 |
