# Supplementary figures and images for: Heat stress-responsive transcriptome analysis in heat susceptible and tolerant wheat (Triticum aestivum L.) by using Wheat Genome Array
Source: BMC Genomics. 2008 Sep 22;9:432. doi: 10.1186/1471-2164-9-432 (PMC2614437; doi:10.1186/1471-2164-9-432)

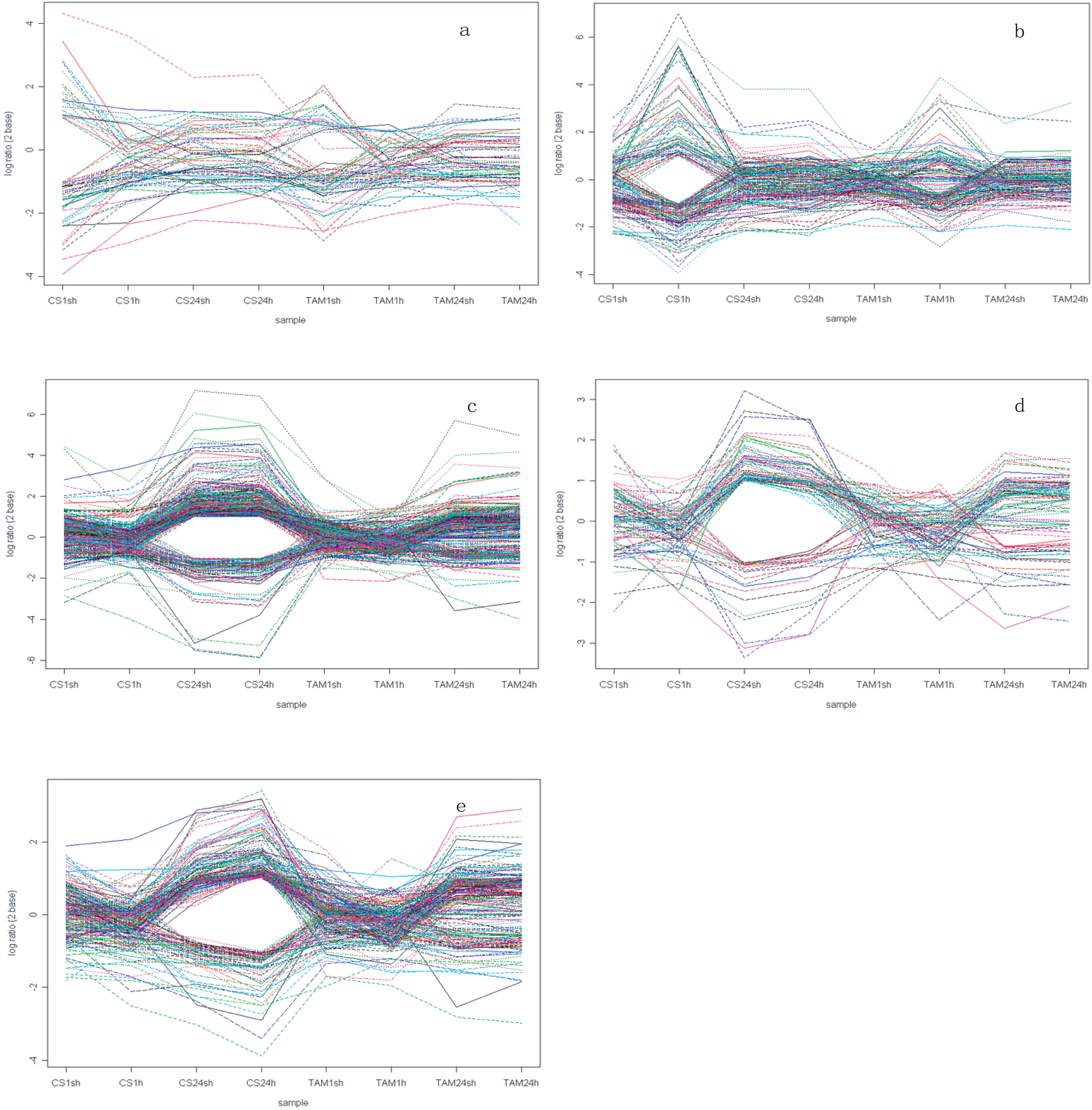

Supplement: Additional file 4 — Expression patterns of the probe sets in Group 2. (a) G2-P3, (b)G2-P4, (c) G2-P5, (d) G2-p6, (e) G2-P7. [file 1471-2164-9-432-S4.jpeg]

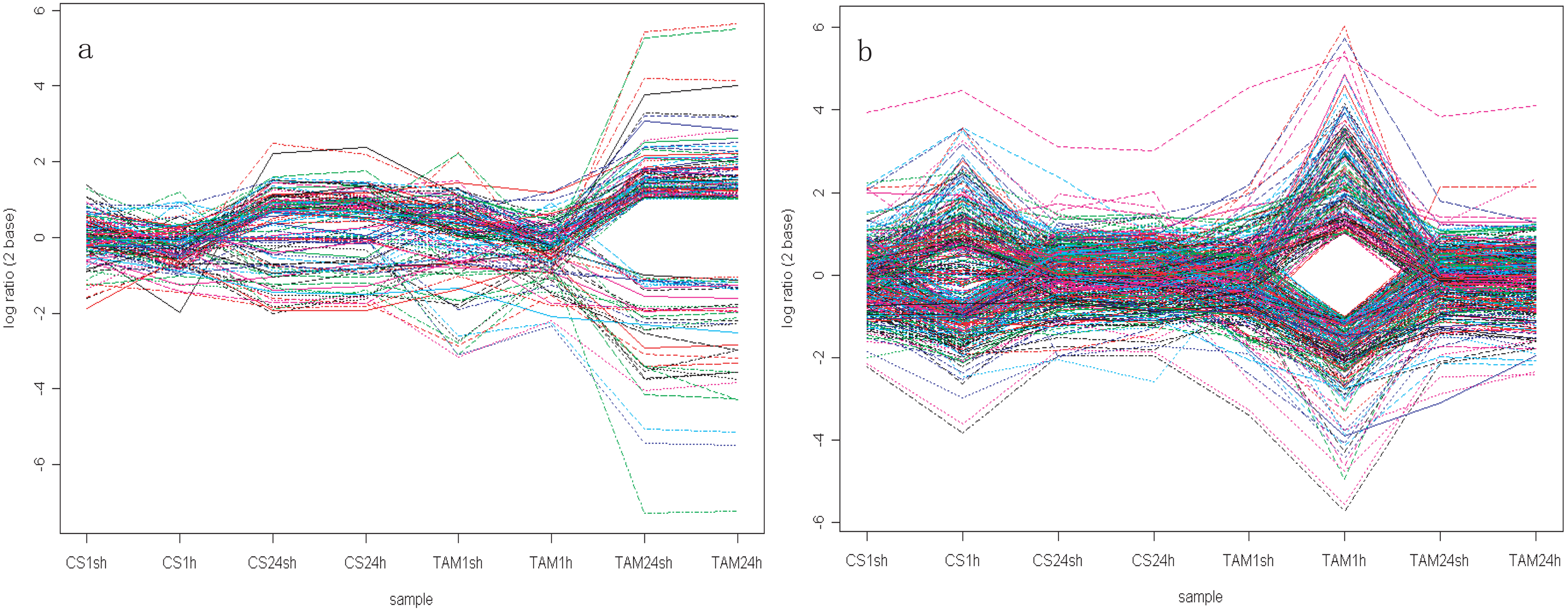

Supplement: Additional file 5 — Expression patterns of probe sets in Group 3. (a) G3-P4, (b) G3-P5. [file 1471-2164-9-432-S5.jpeg]

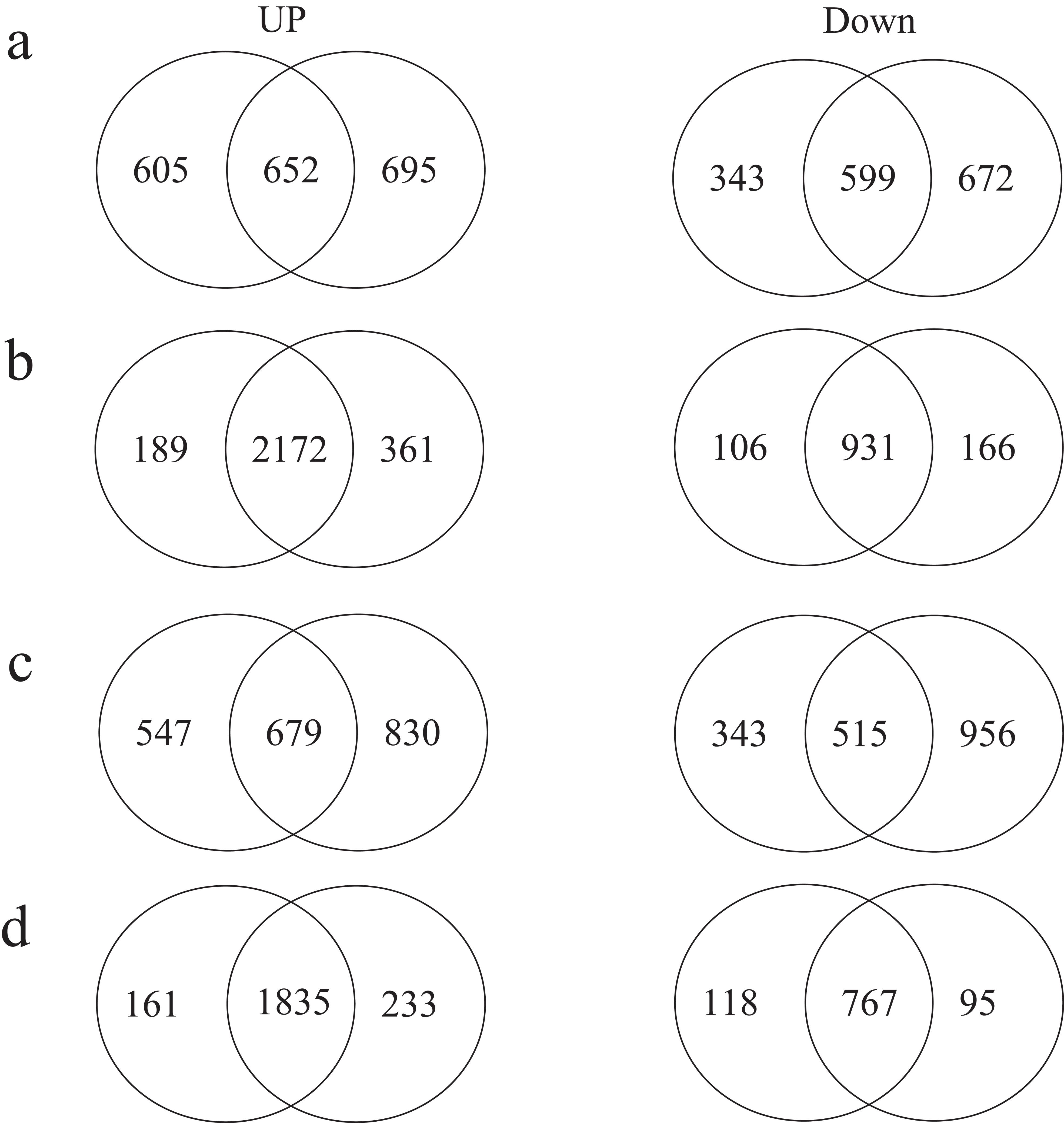

Supplement: Additional file 6 — Overlap of the probe sets regulated in response to heat treatments with and without pre-acclimation. (a) CS1sh (left) and CS1h (right), (b) CS24sh and CS24h, (c) TAM1sh and TAM1h, (d) TAM24sh and TAM24h. [file 1471-2164-9-432-S6.jpeg]

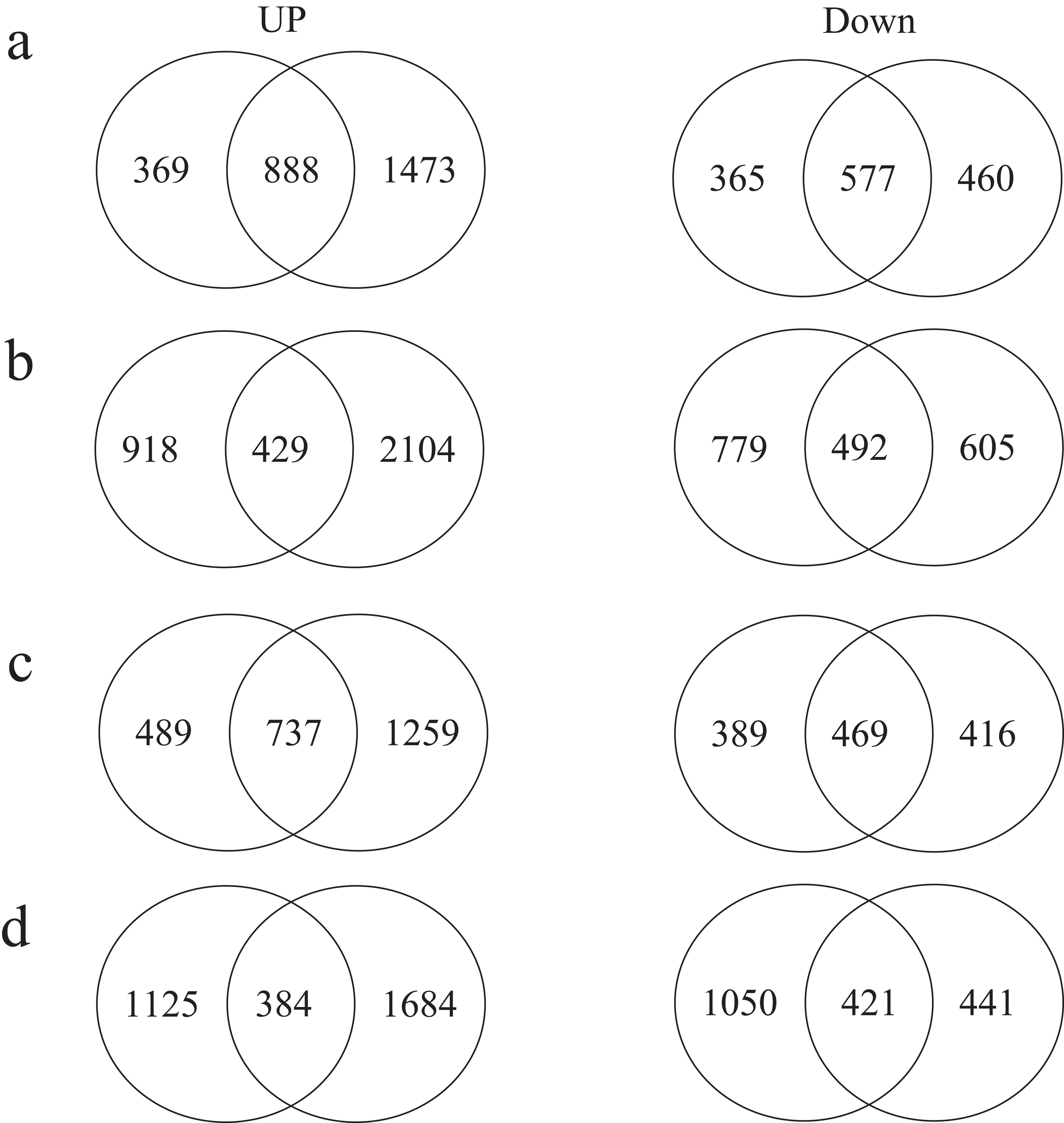

Supplement: Additional file 7 — Overlap of the probe sets regulated in response to 1-h and 24-h heat treatments. (a) CS1sh and CS24sh, (b) CS1h and CS24h, (c) TAM1sh and TAM1h, (d) TAM1h and TAM24h. [file 1471-2164-9-432-S7.jpeg]
