# Supplementary material for: Heat stress-responsive transcriptome analysis in heat susceptible and tolerant wheat (Triticum aestivum L.) by using Wheat Genome Array
Source: BMC Genomics. 2008 Sep 22;9:432. doi: 10.1186/1471-2164-9-432 (PMC2614437; doi:10.1186/1471-2164-9-432)
Supplement: Additional file 8 — Differentially regulated HR probe sets between 1 hour and 24 hours heat treatments identified by nested F-statistic method. 'U' represents up-regulation, 'D' represents down-regulation, 'NC' represents no change (FDR p < 0.001). [file 1471-2164-9-432-S8.doc]

***Additional file 9: Differentially regulated HR probe sets between 1 hour and 24 hours heat treatments identified by nestF-statistic method.***

|  | CS1sh vs CS24sh | CS1h vs CS24h | TAM1sh vs TAM24sh | TAM1h vs TAM24h |
| --- | --- | --- | --- | --- |
| Up | 179 | 181 | 121 | 163 |
| Down | 32 | 19 | 24 | 25 |
| 1-U & 24-D | 5 | 21 | 7 | 37 |
| 1-D & 24-U | 10 | 70 | 16 | 113 |
| 1-U & 24-NC | 142 | 723 | 247 | 910 |
| 1-D & 24-NC | 136 | 393 | 143 | 574 |
| 1-NC & 24-U | 731 | 1782 | 595 | 1395 |
| 1-NC & 24-D | 120 | 237 | 125 | 188 |
| Total Number | 1355 | 3426 | 1278 | 3405 |

“U” represents up-regulation, “D” represents down-regulation, “NC” represents no change (FDR p<0.001)
