# Supplementary material for: Heat stress-responsive transcriptome analysis in heat susceptible and tolerant wheat (Triticum aestivum L.) by using Wheat Genome Array
Source: BMC Genomics. 2008 Sep 22;9:432. doi: 10.1186/1471-2164-9-432 (PMC2614437; doi:10.1186/1471-2164-9-432)
Supplement: Additional file 9 — Expression patterns of HR probe sets representing genes in hormone biosynthesis and signaling pathway. [file 1471-2164-9-432-S9.doc]

***Additional file 6: Probe sets involved in hormone biosynthesis and signaling pathway.***

| ID | CS1sh | CS1h | CS24sh | CS24h | TAM1sh | TAM1h | TAM24sh | TAM24h |
| --- | --- | --- | --- | --- | --- | --- | --- | --- |
| **Ethylene** |  |  |  |  |  |  |  |  |
| ACC oxidase |  |  |  |  |  |  |  |  |
| Ta.22475.1.S1_at | -1.26 | 0.49 | -0.73 | -0.90 | -2.27 | 0.91 | -1.19 | -1.34 |
| Ta.425.1.S1_s_at | -0.33 | -0.04 | -1.73 | -1.82 | 1.14 | -0.05 | 0.06 | -0.21 |
| Ta.9107.1.S1_x_at | -2.53 | -2.58 | -2.47 | -2.45 | -3.56 | -3.44 | -2.70 | -2.58 |
| Ta.9107.2.S1_a_at | -2.55 | -2.56 | -2.40 | -2.78 | -3.68 | -2.90 | -2.74 | -2.85 |
| Ta.9107.2.S1_at | -2.59 | -3.24 | -2.18 | -2.47 | -3.83 | -3.74 | -2.36 | -2.39 |
| TaAffx.100446.1.S1_at | -1.49 | -1.93 | -3.09 | -3.44 | -2.29 | -1.10 | -3.11 | -2.71 |
| TaAffx.80306.1.S1_at | -0.36 | 1.77 | -0.65 | -1.04 | -1.15 | 2.26 | -0.45 | -0.16 |
| ACC synthases |  |  |  |  |  |  |  |  |
| TaAffx.70620.1.S1_at | -1.64 | -1.31 | -1.47 | -1.36 | -1.01 | -1.27 | -1.57 | -1.12 |
| MAPK |  |  |  |  |  |  |  |  |
| Ta.12668.1.A1_at | -0.10 | 0.47 | -1.09 | -0.82 | 0.29 | 0.74 | -0.62 | -0.64 |
| Ta.13373.2.S1_a_at | -0.74 | -0.67 | 1.22 | 1.01 | -0.78 | -1.04 | 0.36 | 0.33 |
| Ta.1715.1.A1_at | -1.05 | -0.92 | -1.39 | -1.42 | -0.30 | -0.47 | -0.65 | -0.61 |
| Ta.25413.1.A1_s_at | -1.84 | -0.85 | -1.58 | -1.69 | -1.74 | -1.04 | -1.13 | -1.15 |
| Ta.3070.1.S1_a_at | -1.76 | -2.31 | -1.41 | -1.23 | -1.98 | -2.42 | -1.74 | -1.62 |
| Ta.3070.1.S1_x_at | -1.80 | -2.20 | -1.38 | -1.25 | -1.69 | -2.28 | -1.54 | -1.59 |
| Ta.4348.1.A1_at | 0.35 | 1.30 | 0.19 | -0.03 | 0.27 | 1.39 | 0.27 | 0.15 |
| Ta.6053.1.S1_at | -1.33 | -0.10 | -1.71 | -1.46 | -1.07 | 0.24 | -1.63 | -1.47 |
| Ta.7205.1.S1_at | -1.29 | -0.69 | -1.69 | -1.51 | -1.11 | -0.57 | -1.46 | -1.44 |
| Ta.7205.2.S1_a_at | -1.81 | -0.56 | -1.99 | -2.20 | -0.95 | -0.34 | -1.63 | -1.60 |
| TaAffx.103414.1.S1_at | -0.59 | -0.88 | -1.19 | -1.22 | -0.14 | -0.86 | -0.92 | -0.95 |
| TaAffx.120620.1.S1_x_at | -1.62 | -2.02 | -0.91 | -1.14 | -0.94 | -0.79 | -0.92 | -0.76 |
| CTR1 kinase |  |  |  |  |  |  |  |  |
| Ta.20406.1.A1_at | -1.37 | -0.98 | -1.67 | -1.83 | -1.34 | -1.19 | -1.57 | -1.59 |
| **Auxin** |  |  |  |  |  |  |  |  |
| 26S proteasome regulatory subunit | | |  |  |  |  |  |  |
| Ta.30743.1.S1_at | 0.56 | 0.02 | 1.18 | 1.33 | 0.69 | -0.20 | 1.25 | 1.24 |
| Ta.766.1.S1_at | 0.81 | 0.93 | 0.65 | 0.61 | 0.89 | 1.35 | 0.42 | 0.51 |
| TaAffx.48202.1.S1_at | 1.73 | 2.88 | 1.63 | 1.62 | 0.95 | 3.42 | 1.33 | 0.96 |
| auxin-responsive factor |  |  |  |  |  |  |  |  |
| Ta.2593.2.S1_x_at | -0.97 | -1.33 | -1.45 | -1.17 | -0.76 | -1.46 | -1.14 | -1.15 |
| Ta.2593.3.S1_x_at | -1.03 | -1.36 | -1.36 | -1.47 | -0.98 | -1.50 | -1.31 | -1.39 |
| Ta.8513.1.A1_at | -0.02 | -0.62 | -0.09 | -0.42 | 0.17 | -1.20 | -0.04 | 0.04 |
| Ta.8537.1.S1_s_at | -1.07 | -1.62 | -0.54 | 0.00 | -0.99 | -2.17 | -0.42 | -0.55 |
| Ta.8605.1.A1_at | -0.26 | -0.52 | 1.11 | 1.62 | -1.09 | -1.37 | 0.32 | 0.11 |
| Ta.8720.3.S1_a_at | -0.92 | -1.54 | -0.43 | -0.37 | -0.80 | -2.32 | -0.59 | -0.56 |
| Ta.9398.1.S1_at | -0.35 | -0.45 | -1.04 | -1.15 | 0.08 | 0.31 | -0.48 | -0.99 |
| TaAffx.122818.1.S1_at | -1.34 | -1.44 | -1.30 | -1.53 | -0.96 | -1.19 | -1.14 | -1.28 |
| TaAffx.34454.1.S1_at | 3.10 | 5.44 | 0.63 | 0.65 | 4.09 | 6.11 | 1.43 | 1.35 |
| TaAffx.59021.1.S1_at | -1.18 | -2.36 | -0.97 | -0.57 | -1.34 | -2.03 | -0.93 | -0.93 |
| AUX/IAA family protein |  |  |  |  |  |  |  |  |
| Ta.10032.1.S1_a_at | -0.41 | 0.09 | -1.05 | -1.08 | -0.41 | -0.26 | -1.05 | -1.12 |
| Ta.10395.1.S1_a_at | -1.05 | -1.07 | -1.44 | -1.57 | -1.60 | -1.40 | -1.84 | -1.68 |
| Ta.10395.1.S1_x_at | -0.80 | -1.13 | -1.49 | -1.57 | -1.70 | -1.45 | -1.81 | -1.73 |
| Ta.10395.2.S1_x_at | -0.87 | -1.22 | -1.40 | -1.51 | -1.66 | -1.43 | -1.81 | -1.82 |
| TaAffx.27045.1.S1_at | -2.37 | -2.33 | -2.21 | -2.15 | -2.35 | -2.10 | -2.06 | -1.78 |
| TaAffx.27045.3.S1_s_at | -2.29 | -2.22 | -1.97 | -2.24 | -1.82 | -2.04 | -1.68 | -1.59 |
| TaAffx.65079.1.A1_x_at | -2.09 | -2.07 | -1.96 | -2.00 | -2.27 | -2.13 | -1.77 | -1.75 |
| TIR1-like |  |  |  |  |  |  |  |  |
| Ta.21131.1.S1_x_at | 0.62 | 0.01 | 1.48 | 1.92 | -0.49 | -0.25 | 0.99 | 0.49 |
| Ta.21131.3.S1_a_at | 1.29 | 1.37 | 1.98 | 2.14 | 0.76 | 1.01 | 1.44 | 1.26 |
| Ta.23215.1.S1_at | -0.94 | -2.52 | -0.23 | -0.55 | -0.65 | -3.13 | -0.32 | -0.49 |
| TaAffx.46586.1.S1_at | -1.59 | -1.35 | -1.20 | -0.91 | -1.46 | -1.41 | -1.10 | -1.01 |
| **GA** |  |  |  |  |  |  |  |  |
| GA3 |  |  |  |  |  |  |  |  |
| Ta.5772.1.A1_at | -0.66 | 0.10 | 0.11 | 0.41 | -1.77 | -1.00 | -1.58 | -1.38 |
| oxidoreductase |  |  |  |  |  |  |  |  |
| Ta.14087.1.S1_at | 2.96 | 1.04 | 3.20 | 3.38 | 1.18 | -0.53 | 1.69 | 1.42 |
| Ta.19011.1.S1_at | -0.24 | -1.48 | 1.26 | 0.79 | -0.50 | -2.03 | 0.07 | 0.49 |
| Ta.9688.1.S1_at | 0.02 | 0.08 | 0.11 | 0.08 | 0.35 | 2.57 | 0.52 | 0.42 |
| RGA |  |  |  |  |  |  |  |  |
| Ta.11110.1.S1_at | -0.88 | -0.67 | -1.05 | -1.54 | -1.31 | -1.13 | -1.15 | -1.31 |
| GAST1 |  |  |  |  |  |  |  |  |
| Ta.11162.1.S1_at | 6.19 | 5.59 | 5.56 | 5.65 | 7.07 | 6.06 | 5.11 | 5.59 |
| **ABA** |  |  |  |  |  |  |  |  |
| ABA-responsive element-binding protein | | | | | | | | |
| Ta.10723.1.A1_at | -1.31 | -1.32 | -1.11 | -1.43 | -1.13 | -0.96 | -0.98 | -0.75 |
| Ta.29331.1.S1_a_at | -0.78 | -1.71 | -0.44 | -0.49 | -0.81 | -2.09 | -0.56 | -0.49 |
| Ta.29331.1.S1_at | -0.75 | -1.53 | 0.23 | -0.02 | -0.77 | -2.58 | -0.24 | -0.27 |
| ABI2 |  |  |  |  |  |  |  |  |
| TaAffx.64499.1.S1_at | 0.07 | 2.88 | 0.00 | -0.01 | 0.02 | 4.80 | 0.00 | 0.00 |
| RAC-like GTP binding protein | |  |  |  |  |  |  |  |
| Ta.23421.1.A1_at | -1.67 | -1.87 | -1.39 | -1.32 | -1.92 | -1.21 | -1.38 | -1.55 |
| Ta.7476.1.S1_at | -1.92 | -2.51 | -1.89 | -1.95 | -2.28 | -2.35 | -1.31 | -1.13 |
| Ta.23421.1.A1_at | -1.67 | -1.87 | -1.39 | -1.32 | -1.92 | -1.21 | -1.38 | -1.55 |
