# Supplementary material for: Heat stress-responsive transcriptome analysis in heat susceptible and tolerant wheat (Triticum aestivum L.) by using Wheat Genome Array
Source: BMC Genomics. 2008 Sep 22;9:432. doi: 10.1186/1471-2164-9-432 (PMC2614437; doi:10.1186/1471-2164-9-432)
Supplement: Additional file 10 — Primer pairs for qRT-PCR. [file 1471-2164-9-432-S10.doc]

***Additional file 10: Primer pairs for qRT-PCR.***

| PID | Forward primer (5'-3') | Reverse primer (5'-3') |
| --- | --- | --- |
| Ta-actin | GGAATCCATGAGACCTAC | GACCCAGACAACTCGCAAC |
| Ta.11162.1.S1_at | CCACCGTCTCAAGTCTCAAC | TACAGGCAGCAAACTCACTC |
| Ta.3784.1.S1_a_at | TTACCCACAGAACAAGGTGC | CCATCACTGAACTTTCCCAGG |
| Ta.9718.1.S1_at | CAAGGCGGTGAAGAATGTG | AGCAACTCAGGGAAGACAC |
| Ta.10155.1.A1_at | CAACACGCAACAGTTCCAG | ACTATTCGGTATGTGCAGTTTTAT |
| Ta.23663.1.S1_s_at | AAGGAGAAGGAGGACAAGAAC | AAACGCAATACAGAGACCAG |
| Ta.681.1.S1_at | CAAGTAGTGGACAGATAAGCCA | CCAACAAGTGACAACATACGG |
| Ta.12225.1.S1_x_at | AAGACAGAGGCGAGGAGAAG | CGGAGGCATTCTTGTTCGTC |
| Ta.28630.1.S1_at | AGGACAGGAACGACAAGTGG | CCACACTCGATCAGCAAGAA |
| Ta.24254.1.S1_a_at | GGTATCGTGGAAAGGAATGGAG | ACCCGTGTCGCTAACATAGT |
| Ta.28955.1.S1_at | CCCAAGACACAAACCTCAGAT | ATCACGGCTGGAGTCAAAC |
